# Maternal complications in molecularly confirmed diandric and digynic triploid pregnancies: single institution experience and literature review

**DOI:** 10.1007/s00404-020-05515-4

**Published:** 2020-03-26

**Authors:** D. Massalska, J. Bijok, A. Kucińska-Chahwan, J. G. Zimowski, K. Ozdarska, A. Raniszewska, G. M. Panek, T. Roszkowski

**Affiliations:** 1 Department of Gynecologic Oncology and Obstetrics, Centre of Postgraduate Medical Education, Czerniakowska 231, 00-416 Warsaw, Poland; 2 Department of Genetics, Institute of Psychiatry and Neurology, Sobieskiego 9, 02-957 Warsaw, Poland

**Keywords:** Diandric triploidy, Digynic triploidy, Maternal triploidy, Paternal triploidy, Partial hydatidiform mole, Preeclampsia

## Abstract

**Objectives:**

Assessment of the maternal complications in molecularly confirmed diandric and digynic triploid pregnancies.

**Methods:**

Sonographic features, biochemical results, and clinical presentation were analyzed. Beta-hCG level was controlled after diandric triploidy.

**Results:**

The study included nine diandric and twelve digynic triploid pregnancies at the mean gestational age at diagnosis of 14.9 and 18.0 weeks, respectively (*p* = 0.0391). Mean value of total-hCG was 979 703.6 U/ml in diandric cases and 5 455.4 U/ml in digynic ones (*p* < 0.000). Maternal complications occurred in 88.9% of diandric triploid pregnancies, including: thecalutein cysts (44.4%), hyperemesis gravidarum (44.4%), symptomatic hyperthyreosis (33.3%), early onset gestational hypertension (22.2%) and vaginal bleeding (11.1%). No case of proteinuria, preeclampsia or HELLP syndrome was observed. Only maternal complication observed in digynic triploidy was vaginal bleeding (50.0%). The mean time of beta-hCG normalization after diandric triploid pregnancies was 84 days (range 11–142 days). No case of gestational trophoblastic neoplasia (GTN) was observed.

**Conclusions:**

Maternal complications (except for vaginal bleeding) are associated with diandric triploidy. The relatively low incidence of hypertensive maternal complications and their less severe course in our cohort may be attributed to the earlier prenatal diagnosis. The frequency of GTN after diandric triploidy may be lower than previously reported.

## Introduction

Triploidy is a lethal chromosomal abnormality resulting from an extra haploid chromosome set of maternal or paternal origin [18]. Pregnancy characteristics depend on parental contribution of the extra chromosomes. Typically diandric triploidy (type I) manifests with a relatively well-grown fetus with normal head size or microcephaly and enlarged placenta of cystic appearance. Digynic triploidy (type II) presents with a severely growth-restricted fetus with a relative macrocephaly (asymmetric fetal growth restriction, FGR) and a very small non-cystic placenta. Moreover, variable structural defects may be observed [25].

The majority of triploid pregnancies is miscarried at an early developmental stage. Triploidy occurs in around 0.03% of pregnancies at 10–14 gestational weeks and 0.002% of pregnancies at 16–20 weeks [14, 20]. Triploidy may cause maternal complications such as preeclampsia, hyperthyroidism, gestational trophoblastic neoplasia or vaginal bleeding [17, 29, 38]. However, assessment of the risk for maternal complications depending on the parental origin of triploidy has not been performed to date.

## Objectives

The objective of the study was to assess the sonographic and biochemical characteristics as well as the risk for maternal complications in the molecularly confirmed diandric and digynic triploid pregnancies that survived beyond 13 gestational weeks.

## Material and methods

### Material

All consecutive singleton triploid pregnancies evaluated at our Ultrasound Department in the years 2017–2019 as a part of TRIPLOIDY PROJECT that survived beyond 13 gestational weeks were included. The study group consisted of 21 singleton triploid pregnancies—9 diandric (69, XXY; *n* = 7 and 69, XXX; *n* = 2) and 12 digynic (69, XXY; *n* = 1 and 69, XXX; *n* = 11).

### Methods

We assessed the sonographic features, biochemical results and clinical presentation of triploid pregnancies examined at our institution in the years 2017–2019. All sonographic evaluations were performed by an experienced sonographer (T.R.). Gestational age was determined from the last menstrual period. Invasive diagnosis for genetic testing was carried out in all cases after informed consent.

The data regarding medical history and complications in present pregnancy (vaginal bleeding, vomiting, hypertension, proteinuria, hyperthyroidism and other abnormalities) were collected from a questionnaire filled by the patients. Urine and blood tests were performed (complete blood count [CBC], and creatinine, uric acid, urea, alanine and asparagine aminotransferase, lactate dehydrogenase, total-hCG, thyroid stimulating hormone [TSH], free thyroxine [fT4]) and blood pressure was measured. In order to exclude gestational trophoblastic neoplasms (GTN) after diandric triploid pregnancies complicated by molar changes the beta-hCG level was controlled every 2 weeks till normal values and a single control follow-up measurement in a month to confirm normal results was performed according to the protocol suggested by Coyle et al. [9].

### Tested samples

21 DNA samples (chorionic villi; *n* = 11 and amniocytes; *n* = 10) with molecular diagnosis of triploidy and 42 matching blood samples from the parents were tested for parental origin with Quantitative Fluorescent Polymerase Chain Reaction (QF-PCR). The mean gestational age at genetic testing was 16.7 weeks (range 11.7–26.3 weeks).

### Quantitative fluorescent polymerase chain reaction (QF-PCR)

QF-PCR reaction included amplification of 26 selected microsatellite sequences from chromosomes 13, 18, 21, X and Y. The products were separated by capillary electrophoresis (3130 Genetic Analyser, Applied Biosystems, Waltham, MA, USA) and the results were analyzed by the use of GeneMapper Software 5.0 (Thermo Fisher Scientific, Waltham, MA, USA). The fetal DNA peaks of quantitative relation 1:2, 2:1 and 1:1:1 were compared to the peaks from parental DNA, which allowed for establishment of the parental origin of the double microsatellite sequences (paternal or maternal origin of triploidy).

### Data analysis

Statistical analysis was performed using STATA 12 (StataCorp). Descriptive statistics was presented with means and proportions. Student’s test and Fisher’s exact test were used to assess differences in continuous data.

### Literature review

We conducted a literature review on the studies describing maternal complications in triploid pregnancies published between 1960 and 2019. Pubmed database (https://pubmed.com) was searched using the term “TRIPLOIDY” and all abstracts were viewed by two authors (DM and AKC). Only full text English-written manuscripts were included. Additionally, the references of all selected manuscripts were screened for subsequent reports. Discrepancies between authors were resolved through discussion, until consensus was reached.

## Results

The study included nine diandric and twelve digynic triploid pregnancies diagnosed at a single institution in the period of three years. Genetic testing was performed at a mean gestational age of 14.9 weeks (range 11.7–17.6 weeks) in diandric cases and at 18.0 weeks (range 12.4–26.3 weeks) in digynic ones (*p* = 0.0391). The mean maternal age at diagnosis in diandric triploidy was 31.1 years and in digynic triploidy: 33.4 (*p* > 0.05).

In all cases the indication for genetic testing was abnormal fetal ultrasound (*n* = 21). Sonographic abnormalities detected in diandric and digynic cases are presented in Table 1. In all diandric cases the placenta was cystic (*n* = 9) and in none of digynic (*n* = 12).

**Table 1:** Sonographic abnormalities in molecularly confirmed diandric and digynic triploidy

Sonographic abnormalities	Diandric triploid pregnancies (*n* = 9)	Digynic triploid pregnancies (*n* = 12)
Fetal growth restriction (FGR)	0 (0%)	11 (100%) All asymmetrical
Oligohydramnios	1 (11.1%)	1 (8.3%)
Structural defects		
Structural defects	8 (88.9%)	10 (83.3%)
Multiple structural defects	8 (88.9%)	7 (58.3%)
Central nervous system defects	5 (55.6%)	5 (41.7%)
Ventriculomegaly	1	1
Enlarged cysterna magna	2	1
Cerebellar malformations	1	1
Holoprosencephaly	1	2
Cardiac defects	6 (66.7%)	6 (50.0%)
Common atrioventricular cannal	1	1
Undefined cardiac defect	3	2
Cardiomegaly	1	
Ventricular septal defect	1	
Tetralogy of Fallot	1	3
Urinary system defects	0 (0%)	0 (0%)
Limb defects	3 (33.3%)	5 (41.7%)
Club foot	2	
Polidactyly	3	5
Other structural defects		
Omphalocele	4	1
Ascites	2	
Hydrothorax	1	
Double buble		1
Hepatolegaly	1	
Facial cleft	1	
Micrognathia		2
Soft sonographic markers of chromosomal abnormalities		
Increased nuchal translucency	6 (66.7%)	0 (0%)
Increased nuchal fold	3 (33.3%)	0 (0%)
Hyperechogenic bowel	1 (11.1%)	0 (0%)
Placental abnormalities		
Cystic placenta	9 (100%)	0 (0%)

The mean total-hCG level at invasive testing in diandric cases was 979, 703.6 U/ml (range 382, 261.0–1, 636, 562.0 U/ml) compared to 5 455.4 U/ml in digynic ones (range 1, 245.2–19, 050.6 U/ml) (*p* < 0.000). CBC, creatinine, uric acid, urea and lactate dehydrogenase levels were normal in all diandric and all digynic cases. In two diandric cases alanine and asparagine aminotransferase levels were slightly increased (2/9; 22.2%).

Maternal complications were observed in eight diandric triploid pregnancies (8/9; 88.9%) compared to six digynic ones (6/12; 50.0%) (*p* > 0.05). Maternal complications in diandric triploid pregnancies included the following: thecalutein cysts: (4/9; 44.4%), hyperemesis gravidarum: (4/9; 44.4%), symptomatic hyperthyreosis: (3/9; 33.3%), early onset gestational hypertension: (2/9; 22.2%) and vaginal bleeding: (1/9; 11.1%) (Table 2). No case of proteinuria, preeclampsia or HELLP syndrome was observed. The only maternal complication observed in digynic triploidy was vaginal bleeding, which occurred in six cases (6/12; 50.0%).

**Table 2:** Characteristics and frequency of maternal complications in molecularly confirmed diandric and digynic triploid pregnancies

	Diandric triploid pregnancies (*n* = 9)	Digynic triploid pregnancies (*n* = 12)
Maternal age (mean; years)	31.1 (range 27.0–36.2)	33.4 (range 26.3–46.1)
GA at genetic testing (g.w.)	14.8 (range 11.7–17.6)	18.0 (range 12.4–26.3)
Pregnancy duration (mean; g.w.)	16.3 (range 14.1–19.6)	19.3 (range 13.4–30.3)
Serum HCG level (mean; U/ml)	979 703.6 (range 382 261.0–1 636 562.0)	5 455.4 (range 1 245.2–19 050.6)
Molar placenta	100%	0%
Thecalutein cysts in ovaries	44.4%	0%
Vaginal bleeding	11.1%	50.0%
Hyperemesis gravidarum	44.4%	0%
Symptomatic hyperthyreosis	33.3%	0%
Gestational hypertension	22.2%	0%
Preeclampsia	0%	0%

*GA* gestational age, *g.w.* gestational weeks

The pregnancy outcome was known for all cases. The mean pregnancy duration was 16.3 gestational weeks in diandric cases (range 14.1–19.6 weeks) and 19.3 weeks in digynic cases (range 13.4–30.3) (*p* = 0.0613). Seven diandric and seven digynic triploid pregnancies were terminated; two and five cases, respectively, ended in intrauterine fetal demise. The follow-up of patients ranged between 6 months and 2 years. In none case GTN was observed. The mean time of normalization of beta-hCG in diandric cases was 84 days (range 11–142 days) and the control measurement after a month was within normal limits in all nine cases.

## Discussion

We present a cohort of consecutive diandric and digynic triploid pregnancies with molecular confirmation that survived beyond 13 gestational weeks. To our knowledge this is the first assessment of maternal complications in such a group of patients. Second trimester triploidy is rare. Nevertheless the size of our study group enables us to draw some conclusions regarding the maternal risks associated with diandric and digynic triploidy, providing a basis for clinic and prenatal genetic counseling.

Molecular confirmation of parental origin was performed in only a few of the triploid pregnancies reported in the literature. Moreover, there is a possible bias provoked by the fact that only most severe cases are reported and their course is not representative for the whole group. The risk for maternal complications was also estimated in two bigger cohorts of triploid pregnancies, but without definitive distinction between diandric and digynic cases [19, 29]. Furthermore, there is a high discrepancy between the results presented in those studies.

The most typical complication of diandric triploid pregnancies is preeclampsia with an early onset before 20 gestational weeks [1, 3, 4, 8, 10, 11, 13, 15, 19, 23, 24, 26, 28, 29, 33–35, 37, 38, 41]. 43 cases of hypertension or preeclampsia in apparently diandric triploidy were reported in the literature with the mean time of onset at 19 gestational weeks (range 14–39 gestational weeks) (Table 3).

**Table 3:** Maternal complications in triploid pregnancies with suspected double paternal contribution

Study	No. of cases, *n*	GA (weeks)	Molar placenta, *n*	Thecalutein cysts of ovaries, *n*	Vaginal bleeding, *n*	Hyperemesis gravidorum, *n*	Symptomatic hyperthyroidism, *n*	GH, *n*	Preeclampsia, *n*	Other complications
Beischer et al. [3]	1	24	1	–	1	–	–	–	1	Cardiomyopathy
Edwards et al. [12]	1	32	1	–	–	–	–	–	–	–
Schindler et al. [30]	1	39	1	–	–	–	–	–	1	–
Paterson et al. [26]	2	27	2	–	1	–	–	–	2	–
Hashimoto et al. [16]	1	22	1	–	1	–	–	–	1	–
Walker et al. [39]	2	36	2	–	–	1	–	–	1	–
Sicuranza et al. [34]	1	18	1	–	1	–	–	–	1	Eclampsia
Wertelecki et al. [40]	1	30	1	–	–	1	–	–	–	–
Lewis et al. [23]	1	21	1	1	–	–	–	–	1	–
Broekhuizen et al. [5]	1	23	1	1	–	1	–	–	1	–
Chatterjee et al. [6]	2	26–27	2	–	2	–	–	–	–	–
Graham et al. [15]	5	16–27	4	1	2	1	–	–	1	–
Pircon et al. [27]	6	18–24	4	2	–	–	–	–	–	–
Cox et al. [8]	5	15–21	4	2	1	1	–	–	5	–
Slattery et al. [35]	1	17	1	–	1	1	–	–	1	Eclampsia
Sorem et al. [36]	1	26	1	–	–	–	–	–	1	–
Jauniaux et al. [19]	20	13–25	20	6	10	4	–	–	3	–
Rijhsinghani et al. [29]	7	15–22	6	–	–	–	–	1	5	–
Ludwig et al. [24]	1	14	1	1	1	–	1	–	1	Severe OHSS
Craig et al. [10]	1	17	1	–	–	–	–	–	1	HELLP syndrome
Rahimpanah et al. [28]	2	16–20	2	–	1	2	1	–	2	–
Daniel et al. [11]	6	16–22	5	–	–	–	–	1	–	–
Stefos et al. [37]	1	18	1	–	–	–	–	–	1	HELLP syndrome
Billieux et al. [4]	1	18	1	–	1	–	–	–	1	Cardiomyopathy pulmonary edema
Barsoom et al. [2]	1	20	1	–	1	–	–	–	1	–
Sherer et al. [33]	1	17	1	–	–	–	–	–	1	HELLP syndrome
Wong et al. [41]	1	17	1	1	–	1	–	–	1	–
Falkert et al. [13]	1	15	1	–	–	–	–	–	1	HELLP syndrome
Anev et al. [1]	1	15	1	–	1	1	–	–	1	Cardiomyopathy pulmonary edema
Uzun et al. [38]	1	16	–	1	–	1	1	–	1	–
Present study	9	14–19	9	4	1	4	3	2	–	–

*GA* gestational age, *GH* gestational hypertension, *OHSS* ovarian hyperstimulation syndrome, *HELLP* hemolysis, elevated liver enzymes, low platelets

The incidence of hypertension or preeclampsia was estimated for 4.3–35% [19, 29]. In our cohort of diandric triploidies hypertension occurred in 22.2% of cases. No case of preeclampsia, eclampsia, HELLP syndrome or cardiomyopathy was noted. The relatively low incidence of hypertensive maternal complications and their less severe course in presented cohort may be attributed to the earlier prenatal diagnosis of diandric triploidy and termination of pregnancies before the onset of life-threatening maternal conditions. Previous cohort studies on the frequency of maternal complications in triploidy were published in the 90s, when the availability of ultrasound was much lower [19, 29]. In the era of broad application of the first trimester screening, the majority of diandric triploid cases may be detected before 14 gestational weeks, as was in our cohort [22]. In 66.7% of presented cases nuchal translucency exceeded 3.5 mm and in all cases with available free beta-hCG results in the first trimester, its level was very high, which resulted in a high patient-specific risk for trisomy 21 and further genetic testing that enabled appropriate diagnosis.

Despite the very high levels of total-hCG that were present in all our diandric cases, symptomatic hyperthyreosis occurred in only 33.3%. We did not observe any relationship of thyreotoxicosis with the total-hCG level, which makes it difficult to predict. Hyperemesis gravidarum and thecalutein cysts were present in 44.4% of cases, respectively. In comparison, Jauniaux et al. reported a much lower rate of thecalutein cysts (8.6%) and hyperemesis gravidarum (5.7%). However, the authors included both diandric and digynic triploid cases to their study (1996). All of our diandric cases had molar changes in the placenta, while only 73% of molecularly confirmed diandric cases described in the literature had molar appearance [21]. Bleeding was reported the most frequent complication in triploid pregnancies [19]. In our cohort bleeding occurred in 50.0% of digynic cases and only in 11.1% of diandric ones.

The frequency of GTN associated with triploid pregnancies beyond 13 weeks of gestation is difficult to establish. Single cases or small series are reported [7, 32]. In a huge group of 265 molecularly confirmed diandric triploid pregnancies with an over 6-month follow-up, no case of GTN was reported. However, gestational age of presented cases was not mentioned in the paper [31]. In our cohort of triploid pregnancies that survived beyond 13 gestational weeks (diandric as well as digynic), with a follow-up between 6 months and 2 years, no case of GTN was observed either. We concluded, therefore, that frequency of GTN after partial hydatidiform mole (diandric triploidy) may be much lower than previously reported [17]. However, the time of beta-hCG normalization after diandric triploid pregnancies may be very long—even exceeding 20 weeks as in one of our cases.

Even though our group consists of all consecutive cases of triploidy evaluated in a single institution, only cases with abnormal ultrasound presentation are reported, which is a limitation of our study. Nevertheless, to our knowledge this is the first analysis of maternal complications in molecularly confirmed diandric and digynic triploid pregnancies and our study may provide a basis for clinic and prenatal genetic counseling.
